# *CrowdPhase*: crowdsourcing the phase problem

**DOI:** 10.1107/S1399004714006427

**Published:** 2014-05-23

**Authors:** Julien Jorda, Michael R. Sawaya, Todd O. Yeates

**Affiliations:** a UCLA–DOE, Institute for Genomics and Proteomics, 611 Charles Young Drive East, Los Angeles, CA 90095, USA; b UCLA, Molecular Biology Institute, 611 Charles Young Drive East, Los Angeles, CA 90095, USA; c Department of Chemistry and Biochemistry, University of California, 611 Charles Young Drive East, Los Angeles, CA 90095, USA

**Keywords:** *CrowdPhase*, crowdsourcing, phase problem

## Abstract

The human mind innately excels at some complex tasks that are difficult to solve using computers alone. For complex problems amenable to parallelization, strategies can be developed to exploit human intelligence in a collective form: such approaches are sometimes referred to as ‘crowdsourcing’. Here, a first attempt at a crowdsourced approach for low-resolution *ab initio* phasing in macromolecular crystallography is proposed. A collaborative online game named *CrowdPhase* was designed, which relies on a human-powered genetic algorithm, where players control the selection mechanism during the evolutionary process. The algorithm starts from a population of ‘individuals’, each with a random genetic makeup, in this case a map prepared from a random set of phases, and tries to cause the population to evolve towards individuals with better phases based on Darwinian survival of the fittest. Players apply their pattern-recognition capabilities to evaluate the electron-density maps generated from these sets of phases and to select the fittest individuals. A user-friendly interface, a training stage and a competitive scoring system foster a network of well trained players who can guide the genetic algorithm towards better solutions from generation to generation *via* gameplay. *CrowdPhase* was applied to two synthetic low-resolution phasing puzzles and it was shown that players could successfully obtain phase sets in the 30° phase error range and corresponding molecular envelopes showing agreement with the low-resolution models. The successful preliminary studies suggest that with further development the crowdsourcing approach could fill a gap in current crystallographic methods by making it possible to extract meaningful information in cases where limited resolution might otherwise prevent initial phasing.

## Abstract 2

The idea of attacking the phase problem by crowdsourcing is introduced. Using an interactive, multi-player, web-based system, participants work simultaneously to select phase sets that correspond to better electron-density maps in order to solve low-resolution phasing problems.

## 1. Introduction   

Compared with humans, computers have the capacity to solve problems at much greater speed. There are many problems, however, where computational speed alone is insufficient to find a correct or optimal solution, for example because the parameter space cannot be fully searched in a practical time. In contrast, the human mind can formulate expert knowledge specific for particular problems, providing a capacity to guide more efficient searches, although with more limited processing speed. The power of the human contribution can be multiplied through the efforts of a greater number of individuals. The term ‘crowdsourcing’, which combines the two domains of human and electronic computing, was coined in 2006 (Howe, 2006 ▶) and since then has seen its definition broadened to a wide range of activities involving a network of people. From a scientific point of view, crowdsourcing can be seen as a community-driven system that distributes a problem into multiple discrete tasks performed by humans.

Over the past few years, approaches that combine the power of a computer algorithm with collective intelligence have flourished and have proved to be successful in solving difficult problems in diverse scientific fields (Khatib, Cooper *et al.*, 2011 ▶; Luengo-Oroz *et al.*, 2012 ▶; Good & Su, 2013 ▶; Lakhani *et al.*, 2013 ▶). One of the leading initiatives is *Foldit* (Cooper *et al.*, 2010 ▶), a multiplayer game for protein structure prediction that was recently used to determine the structure of M-PMV protease with the help of a large gaming community (Khatib, DiMaio *et al.*, 2011 ▶). *EyeWire*, another scientific crowd-based game developed at MIT, made news headlines for involving more than 70 000 players to map the brain network and discover neural pathways. Similar projects have been applied to classify RNA families (Gardner *et al.*, 2011 ▶) and biological pathways (Kelder *et al.*, 2012 ▶).

A challenging problem that might benefit from crowdsourcing is the phase problem in X-ray crystallography. In a diffraction experiment, the observed diffraction pattern allows measurement of the amplitudes of the reflection structure factors (as the square root of the intensities) but not their phases. The amplitudes and phases are both needed to reconstruct an electron-density map (by Fourier synthesis) so that a model of the crystallized molecule can be obtained. Indeed, retrieving the phase information constitutes a major problem in determining a molecular crystal structure. Most macromolecular crystal structures present in the PDB (Bernstein *et al.*, 1977 ▶; Berman *et al.*, 2002 ▶) have seen their corresponding phases retrieved *via* experimental methods that utilize the scattering power of heavy atoms or anomalous scattering atoms to perturb the intensities (Hendrickson, 1991 ▶; Rupp, 2009 ▶; Smith & Hendrickson, 2012 ▶) or by molecular replacement in cases where the structure of a homolog already exists (Rossmann, 1990 ▶; Blow, 2006 ▶; Rupp, 2009 ▶; Scapin, 2013 ▶). However, there are scenarios where phase information is difficult to obtain. Methods based on heavy atoms are not always successful, particularly if the accuracy or resolution of the diffraction data is poor.

On the other hand, many efforts have been made to solve the phasing problem *ab initio*, with success in a somewhat limited range of problems. So-called direct methods are most successful when the resolution is at least 1.2 Å and when the number of non-H atoms is not much greater than one or two thousand (Miller *et al.*, 1994 ▶; Sheldrick, 1998 ▶). Prospects for applying direct methods to obtain phases in situations where the resolution limit is more typical of macromolecular crystals (where the image would not show atomic detail) have led to concepts for improving electron-density maps and corresponding phases based on various complex objective functions (Baker *et al.*, 1993 ▶; Colovos *et al.*, 2000 ▶; Holton *et al.*, 2000 ▶; Terwilliger, 2001 ▶; Lunina *et al.*, 2003 ▶). A number of studies have also aimed at obtaining phases *ab initio* using only low-resolution diffraction data, as reviewed by Lunin *et al.* (2012 ▶). These latter include diverse target functions relying on electron-density histograms (Harrison, 1988 ▶; Lunin, 1988 ▶; Lunin *et al.*, 1990 ▶), map connectivity (Lunin *et al.*, 1999 ▶, 2000 ▶) or other measures of statistical likelihood (Lunin *et al.*, 1998 ▶; Petrova *et al.*, 1999 ▶). Regardless of the particular approach, most attacks on the phase problem can be viewed as having two subproblems. One concerns how a high-dimensional space (*i.e.* of phases) can be efficiently searched, while the other concerns how a good solution can be recognized. In the present work, we aimed to test the utility of collective human intelligence in the latter subproblem (*i.e.* recognition). To perform this effectively required considering what kind of approach to the first subproblem (*i.e.* searching) would be most amenable to being coupled to human-driven recognition and selection.

One powerful search-optimization technique is the genetic algorithm (here abbreviated as GA), which mimics the Darwinian evolutionary process in nature (Goldberg, 1989 ▶). The GA starts with a population of randomly generated candidate solutions (or individuals) and allows them to evolve using computational machinery that relies on random mutations, genetic crossovers and selection. One advantage compared with other optimization algorithms such as gradient-based minimization or simulated annealing (Kirkpatrick *et al.*, 1983 ▶; Polak, 1997 ▶) is that the GA iteratively evolves a whole population of solutions rather than a single solution. This behavior potentially favors the simultaneous exploration of multiple good solutions (or partially good solutions) during the process. Secondly, the stochastic nature of the GA makes it less prone to becoming trapped in local optima compared with gradient-based optimization methods. Genetic algorithms have been applied in previous studies to various crystallographic problems, including phase optimization and *ab initio* phasing. In these studies, the objective functions have included the MABS figure of merit (Webster & Hilgenfeld, 2001 ▶; Abdurahman & Purwanto, 2008 ▶), agreement with noncrystallographic symmetry constraints (Miller *et al.*, 2001 ▶) and the skewness of the electron-density distribution (Uervirojnangkoorn *et al.*, 2013 ▶). Here, we hypothesize that human intelligence can be used as a replacement to a programmatic fitness function and integrated into a conventional GA workflow.

Along these lines, we designed *CrowdPhase*, an online multiplayer game seeking to solve the phase problem for low-resolution situations. The power of *CrowdPhase* resides in the stochastic exploration of a high-dimensional search space by combining a GA with a collaborative human effort. Our use of a GA to search phase space follows closely upon earlier work by others (Webster & Hilgenfeld, 2001 ▶; Zhou & Su, 2004 ▶; Immirzi *et al.*, 2009 ▶), while the use of human pattern-recognition abilities to drive the search represents a new area of exploration. We provide a proof of concept by obtaining *ab initio* phases for two simplified test problems, one containing only two atoms and a second consisting of a short self-assembling polypeptide that forms a hexameric β-barrel or ‘cylindrin’ (Laganowsky *et al.*, 2012 ▶).

## 2. Materials and methods   

### 2.1. Data sets   

To establish the proof of concept of *CrowdPhase*, we generated synthetic structure-factor data for two test problems, so the true phases would be known for the purposes of monitoring and evaluation. The first test problem consisted of two atoms in the unit cell (chosen arbitrarily from the PDB; entry 3sqp, bacterial pyocyanin; Kasozi *et al.*, 2011 ▶). When contoured appropriately, this test case was intended to represent a low-resolution spherical model. Alternatively, a two-atom test case could represent a heavy-atom difference Fourier problem. The second data set corresponds to one of the crystal forms of a 66-amino-acid β-barrel structure (referred to as cylindrin) comprised of six strands packed tightly around a threefold axis of symmetry (PDB entry 3sgn; Laganowsky *et al.*, 2012 ▶). In the second data set, the structure factors were expanded from the cubic space group *I*2_1_3, where the cylindrin assembly sits on a threefold axis of symmetry, to *P*1 using a custom-written script, thereby giving four compact shapes within a *P*1 unit cell. Data sets were subsequently cut to 25 and 18 Å resolution for the two-atom and cylindrin cases, respectively, giving 37 and 67 reflections with independent phases (in *P*1). Test cases were constructed at low resolution in order to provide initial trials suited for human visual analysis. This, and the modest cell sizes, assured limited numbers of variables (*i.e.* phases) for searching in order to provide initial trials favorable for the GA-based search optimization (see §[Sec sec2.5]2.5).

### 2.2. Definition of the starting population   

**Figure 1:**
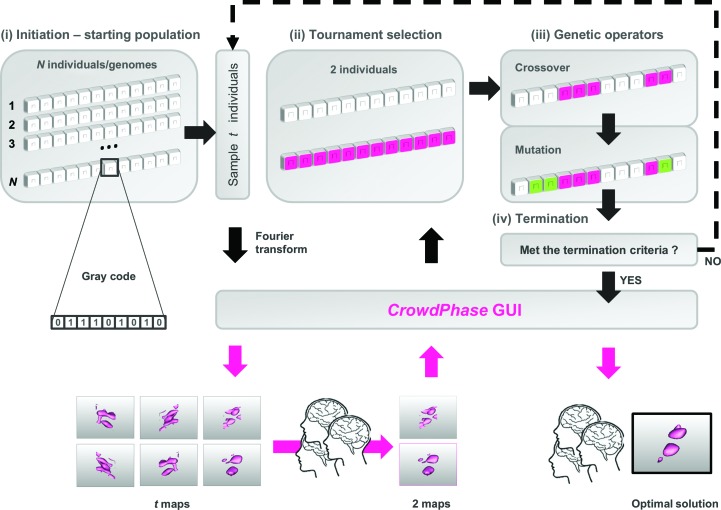
Schematic of the *CrowdPhase* procedure. The *CrowdPhase* core is built on a genetic algorithm that is adapted for human intervention. (i) The GA initiation, where a population of *N* individuals is randomly generated. Each individual is represented as a genome made up of a sequence of genes (pictured as white blocks), each representing a reflection phase. Each gene is itself encoded as a binary 9 bit string. (ii) The population is iteratively evolved through successive rounds of tournaments. During each tournament, individuals are represented in the GUI as electron-density maps from which players are asked to choose two. (iii) *CrowdPhase* accepts the player’s selection and applies the stochastic genetic operators (mutation and crossing over) to this pair, thereby generating one new individual for the next generation. (iv) The population is iteratively replaced by new generations until the termination criteria are met. As a final step, players are asked to choose which map looks the fittest among the last generation, providing *CrowdPhase* with its final solution.

Upon starting a GA run, a first population of solutions (also called individuals) is initialized. In this initial population, all individuals share an identical set of experimental structure-factor amplitudes but different randomly generated phase sets. To adapt the phase problem to the GA formalism, we modeled each individual as a genome, where the phase of one reflection is represented by a gene. In the same vein, the expressed phenotype for a given genome derives from its electron-density map in real space. Each phase can take a discretely sampled value between 0 and 359, modeled as a 9 bit binary string encoded in binary-reflected Gray code. With this encoding, two successive codes (*i.e.* adjacent angular phase values) differ in only one bit position (Fig. 1 ▶), enabling smooth transitions between similar phase values by flipping one bit in the string.

### 2.3. Successive generations   

Each new generation is produced from the previous generation by a series of parental choices made by game players. A game player chooses two parents from the current generation that he or she judges to be highly ‘fit’, based on electron-density appearance. The genomes of these two chosen individuals are then recombined (by random crossover) and randomly mutated to give one new individual in the next generation. This procedure continues until the next generation is fully populated (*i.e.* with the same number of new individuals as in the previous generation).

### 2.4. The weighted r.m.s. phase error   

For problem testing and player evaluation, known (correct) phases are calculated and used for comparison. The error for an individual is taken as the r.m.s. phase difference, weighted by structure-factor amplitudes,
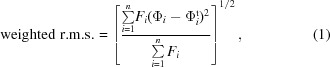
where *n* is the number of reflections or individuals, *F_i_* is the amplitude of structure factor *i*, Φ_*i*_ is the phase of structure factor *i* for the individual in question and Φ^t^_*i*_ is the true phase for structure factor *i*.

In (1), stronger reflections will contribute more to the total r.m.s. phase error than weaker ones. Theoretically, the weighted r.m.s. (WRMS) phase error can take values in the range (0, 180°), where 0° would be associated with an individual having the same phases as the true solution and 180° would correspond to the Babinet representation of the true solution. The expected WRMS phase-error value for random phases is 90°.

### 2.5. Genetic algorithm parameters   

Parameters of the GA are set before the initiation of a run. They include the number of genes (or phases) *n*, the size of the population *N*, the tournament size *t*, the crossover rate *c* and the mutation rate *m*. Since the behavior of the search is highly dependent on these parameters, benchmarks on data sets with known phases were performed to determine suitable values. In particular, we noted that the genome size *n* had a strong impact on the width of the WRMS phase-error distribution across the population. As might be expected, the standard deviation of the WRMS phase-error distribution for a whole population is inversely correlated to the number of reflections encoded. In other words, for larger genomes the standard deviation of the WRMS phase error is smaller. From our observations in test cases, we judged that an average human evaluator was able to discriminate two individuals if their mutual WRMS phase errors differed by at least 5°. We found that this condition was generally satisfied when the number of genes *n* was no greater than about 70. The algorithm parameter *t* defines the number of individuals (out of *N* total) that are randomly sampled to create a single ‘tournament’ selection, from which the two individuals judged to be fittest will be chosen. We found that presenting only a subset of the total individuals to a user in each tournament reduces the perceived difficulty of the selection task without substantially degrading the algorithm performance. Thus, *N* tournaments, each involving *t* individuals sampled from the population, will be necessary to create a new generation. Other important parameters are related to the genetic operators: the crossover and the mutation. We use a uniform crossover probability, where each gene locus is tested against the crossover rate *c*, with the outcome of the test defining which parent will contribute to the offspring at each locus (Fig. 1 ▶). For the subsequent mutation step, each bit of each gene is tested against the mutation rate *m*; if the test succeeds, the corresponding bit is flipped. In general, a combination of *N* = 120, *t* = 12, *n* ≤ 50, *c* = 0.15 and *m* = 0.15 during automatic benchmarks kept the standard deviation of WRMS phase-error values across the population higher than 5° (preventing premature convergence). This yielded fast convergence to good solutions when accurate selection conditions were tested (*e.g.* in automatic trials where the computer was used to make perfect fitness evaluations). The last parameter, called the termination criterion, is defined as a maximum number of iterations (or generations) before stopping the evolutionary process. In this work, we set the number of generations to 20 for all GA runs.

### 2.6. Cross-correlation method   

We developed a calculation, largely inspired by Thomas & Schmid (1995 ▶) and similar to Lunin & Lunina (1996 ▶), to untangle the origin, enantiomorph and Babinet ambiguities that have to be considered before allowing a genetic crossover between the phase sets belonging to two selected individuals. Let *I*_1_ and *I*_2_ be the two individuals to merge, *F*(*h*)_1_ and *F*(*h*)_2_ their respective structure factors and *R*(*h*) their cross-correlation spectrum:
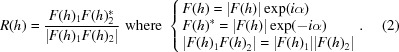
If developing (2), and considering that the amplitudes of both *F*(*h*)_1_ and *F*(*h*)_2_ are the same, then *R* can be written as
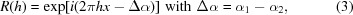
where α_1_ and α_2_ are the phases for *F*_1_ and *F*_2_. Subsequently, the cross-correlation function (CCF) will be formulated as
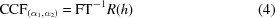
and
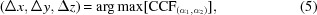
where the CCF is the inverse Fourier transform of *R*(*h*) and (Δ*x*, Δ*y*, Δ*z*) are the coordinates of the peak in the map produced by the CCF. The equation used here for the correlation function is similar in purpose to but slightly different in form compared with the translation-function equations typically used to establish relative shifts (Colman *et al.*, 1976 ▶; Read & Schierbeek, 1988 ▶; Tong, 2006 ▶). At least in cases where good correspondence exists between the two functions, the result is not likely to be sensitive to the particular choice of the form of the correlation function used. From the (Δ*x*, Δ*y*, Δ*z*) coordinates obtained from the calculation, the shifts to be applied to the phases of the individual *I*_2_ to match the phase origin of *I*_1_ can be calculated. The required phase shift has the form exp[2π*i*(*h*Δ*x*)]. Note that the test cases in this study were constructed in *P*1, so all possible relative origin shifts are relevant for comparison. For cases involving space groups with higher symmetry, only shifts corresponding to equivalent origins need to be checked.

The same procedure can be successively applied to investigate which of the enantiomorph and Babinet copies for the individual *I*_2_ is the best to use during the recombination. By our procedure, we implicitly assert that at low resolution the user will not be able to discriminate between correct and incorrect hands of an electron-density map; at much higher resolutions, this assertion would have to be re-examined. Furthermore, the visual examination in our graphical user interface is based on a molecular contour envelope, and numerical negation of the map (*i.e.* the Babinet opposite) produces the same contours. We therefore treat Babinet inversions as essentially indistinguishable in the present study. Particularly when the solvent content is much higher or much lower than 50%, Babinet inversions rendered at appropriate contour levels might be distinguishable, a possibility not investigated here.

The enantiomer and the Babinet versions of *I*_2_ translate into phases equal to −α_2_ and α_2_ + π, respectively. The decision on which phase modification, *i.e.* enantiomer, Babinet, neither or both, should be applied to individual *I*_2_ depends on which of the following four cross-correlation peaks has the largest value: arg max[CCF_(α1,α2)_] (if this is the largest, then no phase modification), arg max[CCF_(α1,−α2)_] (if this is the largest, then negate α_2_), arg max[−CCF_(α1,α2)_] (if this is the largest, then add π to α_2_) or arg max[−CCF_(α1,−α2)_] (if this is the largest, then negate α_2_ and add π).

After modifying one phase set (if required), *I*_1_ and *I*_2_ will be able to mate and generate a new individual according to the genetic operators earlier defined. Which of the two individuals being mated is chosen as the reference and which is modified by the shift (including a possible change in hand and sign) is arbitrary, so the new individual resulting from the mating also has arbitrary hand and sign.

### 2.7. Map generation and the GUI   

**Figure 2:**
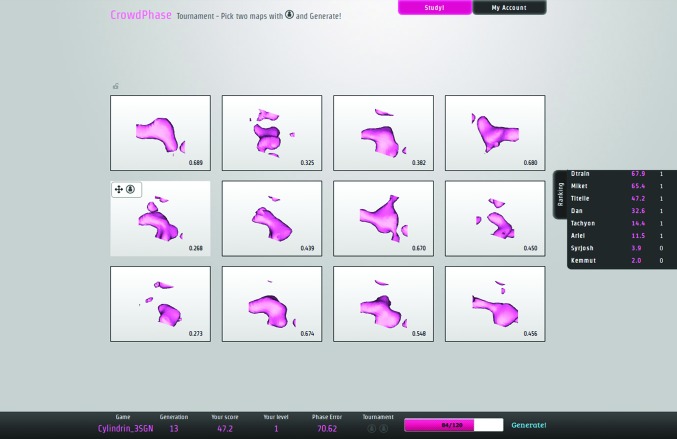
Screenshot of the graphical user interface. A tournament displays a set of electron-density maps randomly sampled from the total population, which is different for each player. The status bar at the bottom of the screen displays information about the genetic algorithm status: the current generation, its average phase error, the progress bar until next generation and the player’s score and level.

For each individual, an electron-density map is generated *via* a direct summation of the Fourier terms. All data sets used in this work were treated in the *P*1 space group. The maps are drawn with a contour level of 1.5σ and surfaces are subsequently rendered by the marching cubes algorithm (Lorensen & Cline, 1987 ▶). The final coordinates of the surfaces are exported to files in Wavefront .obj format. These files are then interpreted with a customized JavaScript engine as three-dimensional objects in the HTML5 canvas element, allowing any modern web browser to display them with no additional plugin. Within the *CrowdPhase* GUI, the maps can be rotated separately or all at once for easier comparison (Fig. 2 ▶).

### 2.8. Access to the *CrowdPhase* gaming system   

The URL for *CrowdPhase* is http://www.crowdphase.com.

## 3. Results   

*CrowdPhase* relies on users at two decisional steps of the genetic algorithm workflow, namely tournament selection and termination. To attract players who may have no crystallo­graphic background, we present the program as a multiplayer game platform, allowing users to interact with the GA *via* a GUI in an intuitive way (Fig. 1 ▶). Within this framework, an *ab initio* phasing puzzle can be seen as a game, and the tournament selection of two parents, here two electron-density maps judged to have desirable features, to propagate a new individual constitutes one round or tournament of the game. As diagrammed in the flowchart in Fig. 1 ▶, many players participating simultaneously populate each new generation, one after the other, until a termination condition is met and game play ends. We used *CrowdPhase* to solve two low-resolution puzzles: a case of two (distantly separated) atoms at 25 Å resolution and a small six-stranded β-barrel (PDB entry 3sgn) at 18 Å resolution. For simplicity, the structure factors were expanded from their original space group to *P*1, resulting in 37 and 67 structure factors for the two-atom and the β-barrel cases, respectively (see §[Sec sec2]2 for details).

### 3.1. Control runs with automated decision making   

As an initial step, we wanted to test whether the GA could reach good phase solutions if driven by an objective target function. We used an automatic version of the GA implemented in *CrowdPhase* with no human intervention. Two objective functions were investigated for both puzzles: one was the WRMS phase error, to simulate the behavior of omniscient users, while the second was a single numerical descriptor of the electron-density distribution asymmetry, the skewness. Positively skewed maps tend to reflect higher quality maps than those having a negative skew (Lunin, 1993 ▶), and skewness has been demonstrated to be useful for improving the quality of maps in the *PHENIX Autosol* wizard (Terwilliger *et al.*, 2009 ▶). More recently, the skewness of the electron-density distribution, coupled to a genetic algorithm, proved to significantly improve low-resolution experimental starting phases (Uervirojnangkoorn *et al.*, 2013 ▶). The skewness of each individual is displayed alongside its electron-density map in the *CrowdPhase* GUI (Fig. 2 ▶).

**Figure 3:**
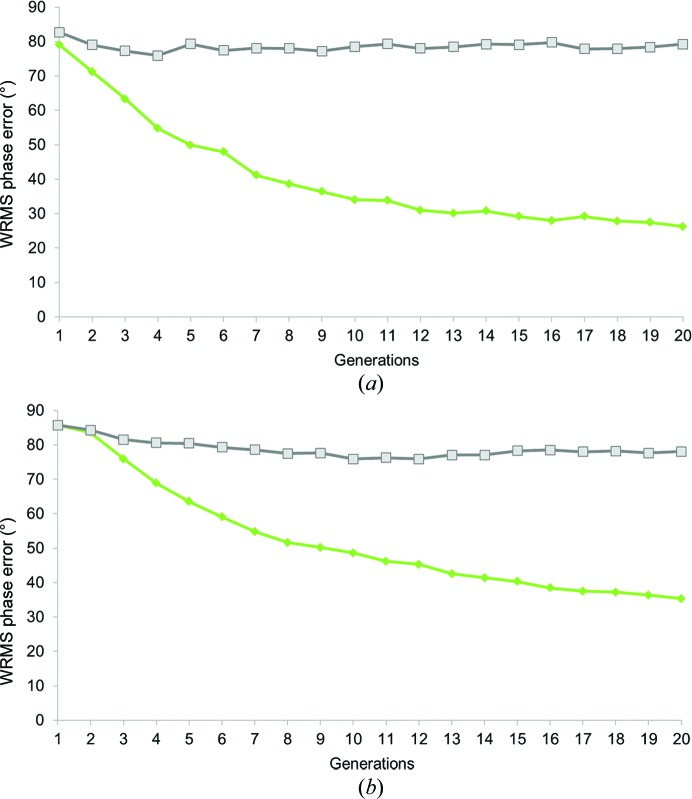
Control cases for performance of the genetic algorithm when run under defined fitness functions. (*a*) Results of running the genetic algorithm on the two-atom case with automatically defined fitness functions. The histogram represents the evolution of the average WRMS phase error for each generation. The two curves illustrate the results of using the WRMS phase error (green) and the skewness (grey) as the fitness function. (*b*) The same control experiment as in (*a*), but applied to the second test case, a small polypeptide assembly.

The automated (nonhuman) GA runs were initiated with the parameters *N* = 120, *t* = 12, *c* = 0.15, *m* = 0.15 and a maximum number of 20 generations as the termination criterion. In each tournament (*i.e.* a presentation of a subset of the current population), the two individuals with the highest calculated fitness scores were subjected to the genetic operators (crossover and mutation) to create a new individual for the next generation. At the end of a run, we assessed the generated solutions. For quality assessment, we used the average WRMS phase error of the whole population for each generation (Fig. 3 ▶).

As expected, the GA simulation runs with the omniscient phase-error-based fitness function converged steadily towards solutions with better phases. The threshold for success, which we set at 30° for the population WRMS phase error, was met after 11 and 16 generations for the two-atom and the polypeptide cases, respectively. The best individuals in the terminal generation had a WRMS phase error of 15° for the two-atom puzzle and 25° for the polypeptide assembly (Fig. 3 ▶). These results constitute a positive control showing that the GA will operate properly with the given parameters when it is guided by sufficiently accurate selection. We noted that for both puzzles the standard deviation never dropped below 5°, which reflects the degree of genetic diversity maintained by our choice of GA parameters, as discussed earlier in §[Sec sec2]2.

The second test, which involved the skewness as a sole objective function, behaved differently. In the early steps (generations 1–3) the quality of the maps seemed to improve slightly, with a decrease in the WRMS phase error of 3 and 5° for the two-atom test and the cylindrin polypeptide test, respectively (Fig. 3 ▶). However, further generations during these GA runs failed to converge towards better solutions; their global WRMS phase errors stagnated until the final generation. This shows that the skewness cannot be used reliably as a sole fitness function for *ab initio* phasing of low-resolution macromolecular data sets. These observations further motivated our idea to adapt the traditional GA workflow in order to employ human decisions as a fitness function in *CrowdPhase* (Fig. 1 ▶). However, since we noted slight improvements during the first steps of the test using only skewness as a fitness function, all *CrowdPhase* experiments in our studies were started with a single initial automatic iteration using skewness.

### 3.2. Program features and game operation   

#### 3.2.1. Initiation of the game   

The procedure initiates with a starting population of *N* randomly generated individuals (*i.e.* random phase sets) and standard GA parameters. The choice of these values is described in §[Sec sec2]2, as is the rationale for operating with a total number of reflection phases (or genes, *n*) less than about 70.

#### 3.2.2. The fitness function and the tournament selection   

The next step, as depicted in Fig. 1 ▶, is the iterative tournament selection, where the fittest individuals are chosen and mated to build a new generation. This is the first level of human commitment compared with a classic GA procedure. A tournament can be seen as a genetic roulette where *t* individuals are randomly sampled from the population, and the two individuals from this group that are judged to be fittest will mate to give birth to a new individual for the next generation. In *CrowdPhase*, the fitness function is the players’ pattern-recognition capabilities (Fig. 1 ▶). To enable systematic display and selection of electron-density maps, we implemented the tournament play within a user-friendly web interface that displays a rectangular array of electron-density maps, one for each of the *t* individuals randomly sampled from the population (Fig. 1 ▶). Each map can be seamlessly rotated and manipulated *via* the GUI (Fig. 2 ▶).

Multiple tournament rounds are independent of each other; different players participating simultaneously see different sets of maps during their separate tournaments. Upon the submission of a player’s two best candidates, the web server recombines the two individuals into a new one for the next generation, after addressing the crystallographic ambiguities inherent to the merging of different phase sets (see §[Sec sec2]2). The total number of tournaments required to complete a new generation is the same as the number of individuals in the population, *N*. The web interface displays the number of new individuals that have been generated for the next generation in real time *via* a progress bar (Fig. 2 ▶). At present, no attempt is made to balance the number of new individuals that are generated by different players. When the next generation has been populated, *CrowdPhase* generates electron-density maps for this new generation of individuals.

#### 3.2.3. Termination and final selection   

Successive generations are produced until the algorithm meets the termination criterion (Fig. 1 ▶). At the termination point, human intelligence is involved in the final selection stage of the GA workflow (Fig. 1 ▶). Players are invited to vote for the best individual from the final population. The final solution is taken to be the individual with the most votes.

#### 3.2.4. Training and user guidance   

Test applications (where the correct phases are known) make it possible to train and monitor player performance. In order to ‘gamify’ the tournament selection and engage the players in a competition, a scoring system was implemented to evaluate the correctness of a player’s tournament selections. In addition, participating players are ranked according to their accumulating score *via* the interface (Fig. 2 ▶).

Because *CrowdPhase* is intended for players that may have no crystallographic background, it is crucial to train the users before they participate in blind (and probably more complicated) cases. With this in mind, for the two test problems that we examined in the present study, we defined two levels for the players. Level 0 is for players who are still practicing. Their tournament choices were not used to drive the GA. Players are then required to reach a threshold score in order to level up to level 1, where their decisions drive the GA. As a score, we took the log base two of two times the probability that individuals chosen randomly would be poorer than the individuals chosen by the player. Note that this scheme tends to give a score of 0 if the user’s choices are effectively random or uninformed, since the chance that a randomly chosen individual would be poorer would be 1/2, and the log_2_ of twice this value is 0. Good choices by a user produce positive values. Under this scheme, we set the threshold at a cumulative score of 5 for a player to advance to level 1. In these first experiments, the training stage for each user was seamless with the active phasing stage; the user’s choices began to contribute to new generations as soon as the score threshold was first passed. In addition to providing feedback scores during training, the user is also able to examine for comparison a set of maps with high phase error and a set of maps with low phase error. This seems to help users recognize features correlated with improved phases. These maps do not represent individuals from the evolving GA population, but rather represent an independent source of study and training. Again, because these test cases involve synthetic data, maps with known amounts of phase error can be calculated.

Finally, we added the skewness of the electron-density distribution as a complementary guide during the tournament selection. The skewness value for each individual is reported alongside its electron-density map by the user interface.

### 3.3. Crowdsourcing performance on test problems   

#### 3.3.1. First game: the two-atom problem   

**Figure 4:**
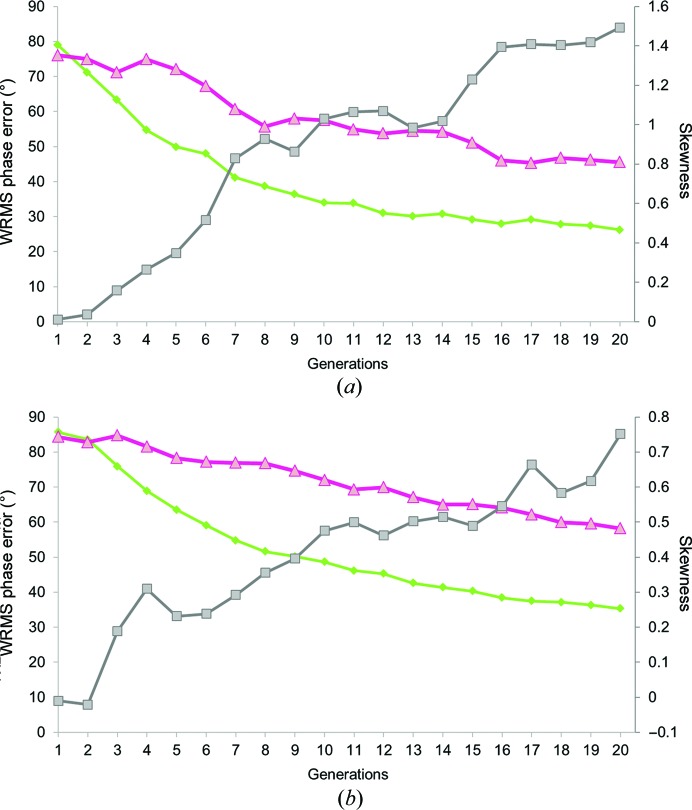
Performance of human crowdsourcing on two low-resolution phasing test cases. Plots showing the evolution of WRMS phase error during a game. In both the two-atom (*a*) and the cylindrin polypeptide (*b*) cases, the human-powered GA drove convergence towards solutions with much lower phase errors than random (pink). For comparison, the trend of the automatic GA using the WRMS phase error as a fitness function is also shown (green). The skewness for the whole population features an inverse correlation to the average WRMS phase error across the generations (grey).

**Figure 5:**
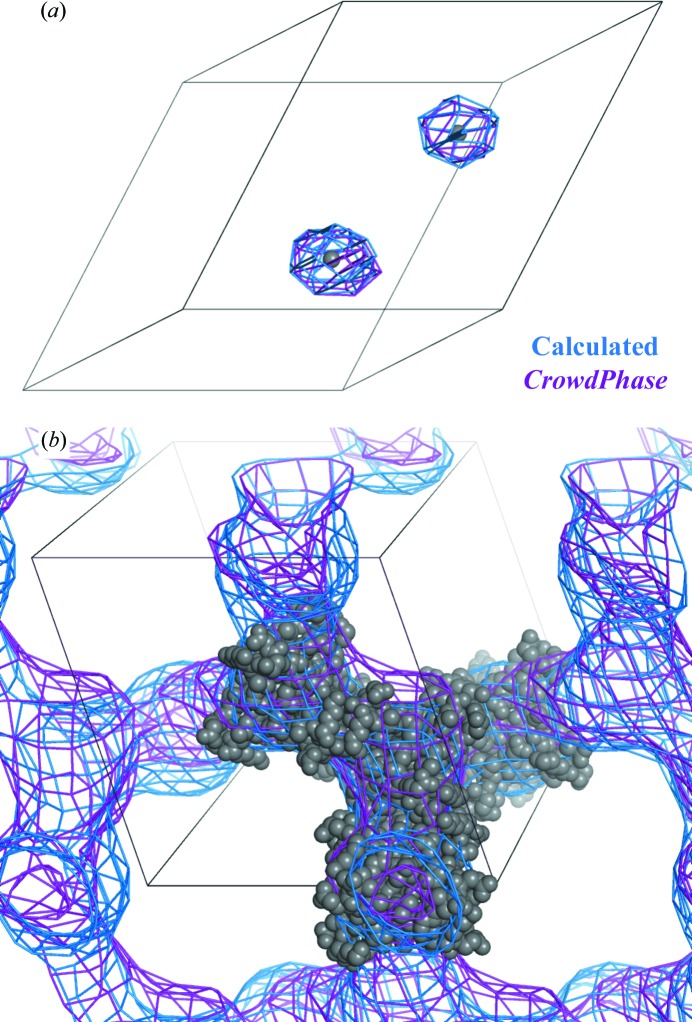
Overlay of electron-density maps calculated from the final crowdsourced phase solutions and the true phases for the two test cases. The maps were generated with phases obtained from the *CrowdPhase* solutions (purple) and the correctly phased structure factors (blue). The two-atom case is shown in (*a*) and the cylindrin polypeptide case is shown in (*b*). Atomic models from the respective PDB entries are shown overlaid on the maps.

This game involved a crowd of 26 people, mostly composed of undergraduate students who did not have any prior training in X-ray crystallography. Upon joining the project, players were introduced to the concept of *CrowdPhase* and were subsequently invited to play the game. Of the 26 users, 18 successfully passed to level 1; the other eight players did not participate actively or make a substantive attempt to advance. Players were aware that the correct phases were for a two-atom structure. Similarly to the control experiments, but now with gamers directing the evolution of the GA *via* the *CrowdPhase* web interface, the initial random population was evolved over 20 generations (one automatically driven by the skewness followed by 19 driven by human players). The progression of the average WRMS phase error for each generation is shown in Fig. 4 ▶(*a*). Overall, the WRMS phase error of each generation substantially decreased, reaching a global WRMS phase error of 45° at the termination step. The fittest individual of the population exhibited a WRMS phase error of 26°. Upon termination, users were asked to choose the best individual from the 20th generation through a vote system. The outcome of the termination step classified the map of the fittest individual at the top of this list, with a total of four votes. Other individuals (maps) receiving votes had WRMS phase errors of 29 and 37°. The average skewness was also monitored for each generation (Fig. 4 ▶), and increased systematically over the generations, although as noted earlier the skewness alone was not a sufficiently specific criterion to drive the system to a good overall set of phases. Fig. 5 ▶(*a*) illustrates the electron-density map calculated with the phases of the optimal solution overlaid with a map calculated with perfect phases. The interpretation of the final model as two compact density features is clear. The map correlation coefficient is 0.91.

#### 3.3.2. Second game: the cylindrin polypeptide case   

This puzzle represents a more realistic application to crystallo­graphic problems. The unit crystal contains a small but bona fide protein complex. The solvent content is about 78%, providing a favorable case for recognizing molecular boundaries in the unit cell. This game involved only 17 initial players ranging from scientists to non-experts, of which 11 participated actively and reached level 1. The users were given no prior information about their target, other than the general concept that the correct solution would likely correspond to a relatively smooth molecular boundary. The same game procedure was followed as for the first test case. The evolution of the average WRMS phase error and the skewness for each of the 20 generations are reported in Fig. 4 ▶(*b*). The WRMS phase error in this case did not improve as rapidly as in the first puzzle. However, at the termination step the best individual in the final generation had a WRMS phase error of 49°. The users voted for the fittest individual, and that consensus choice had a WRMS phase error of 54°. Other choices featured WRMS phase errors ranging from 49 to 58°, which is better than the global phase error of the last generation. The map calculated from this optimal solution had a correlation of 0.85 compared with the map of the true solution. An overlay showing general agreement of the electron-density map generated through *CrowdPhase* with the correct model is pictured in Fig. 5 ▶(*b*).

## 4. Discussion   

As we go further into the social networking era, a picture of how difficult tasks can be tackled by a collaborative intelligence is emerging. Crowdsourcing in science is on the rise, with its feasibility having been demonstrated by several recent projects (Cooper *et al.*, 2010 ▶; Gardner *et al.*, 2011 ▶; Khatib, DiMaio *et al.*, 2011 ▶; Kelder *et al.*, 2012 ▶; Luengo-Oroz *et al.*, 2012 ▶; Good & Su, 2013 ▶; Lakhani *et al.*, 2013 ▶). Our work is the first attempt to bring the crystallography and crowdsourcing fields together, and aims initially at *ab initio* low-resolution phasing. Crowdsourcing relies on the division of a problem into elementary and independent tasks, and we designed *CrowdPhase* with this in mind, using a genetic algorithm as the engine for exploring the high-dimensional space of unknown phases. The parallelizable nature of the tournament selection step makes the GA an ideal candidate for distributive work, and indeed GA-based algorithms have proven to be useful in previous crystallographic search procedures (Webster & Hilgenfeld, 2001 ▶; Zhou & Su, 2004 ▶; Immirzi *et al.*, 2009 ▶; Uervirojnangkoorn *et al.*, 2013 ▶). The novelty here resides in the combination of an efficient search procedure with a non-algorithmic (*i.e.* human) selection criterion. The crowd­sourcing format aims to overcome the limited speed of the human brain by harnessing the power of many users working in parallel.

Two simple games were developed as benchmarks to evaluate performance with non-expert players. One trial involved only two atoms, and a second one used a polypeptide oligomer that forms a compact β-barrel. The effectiveness of the phasing method was evaluated using common measures such as mean phase error or the map correlation coefficient (Lunin & Woolfson, 1993 ▶). As a positive control, we showed that when the phase error was used directly as the fitness function in an automated run, the GA parameters selected were adequate for good convergence towards solutions with low phase error. This demonstrated that the overall approach can succeed under conditions of sufficiently good selection.

With similar GA parameters set in *CrowdPhase*, but basing the fitness function on human intuition, relatively small groups of players succeeded in finding good phase solutions. Indeed, players were able to generate individuals corresponding to interpretable maps even before reaching 20 generations in the first puzzle. Likewise, in the second case the GA run converged towards a solution with a phase error and map correlation coefficient significantly better than random. However, for the second case we note that our target success threshold of 30° was not satisfied after 20 generations. The discrepancy between the final results obtained for the two puzzles can be explained by the fact that the two-atom maps represent two discrete and well defined objects (which was prior information given to the players), as opposed to the connected density blobs encountered in the second case. This also illustrates that in cases where the correct result is not known in advance, making good choices for the GA parameters (such as the number of generations before termination) will present an important challenge. Also, the genome size (*i.e.* the number of reflections to phase) in the cylindrin polypeptide problem was almost twice the number as for the two-atom problem (67 *versus* 37), which affects the efficiency of the GA in both the automatic (Fig. 3 ▶ *b*) and the human-powered (Fig. 4 ▶ *b*) test. For this puzzle, either decreasing the genome size to 50 phases or extending the termination criterion could have been beneficial to the convergence. In Fig. 4 ▶, the slopes of both curves are not as monotonic as when the WRMS phase error was used as the fitness function in the automatic runs (Fig. 3 ▶). This illustrates that players are not perfect in their judgment, and also suggests that performance might be better if users were subjected to more extended training before using their choices to drive the GA. Also, players who join a game in progress can negatively impact the phase-error evolution while they are still improving. In Figs. 4 ▶(*a*) and 4 ▶(*b*), the small fluctuations during the early generations in both puzzles highlights that even after our rudimentary practice phase, players still often falter and make poor choices. Overall, we noted a broad range of skill levels by different players, further emphasizing the value of player training for the performance of the *CrowdPhase* system.

Additionally, our results highlight that the map skewness can be useful for improving the phasing during initial iterations from random starting phases, although it cannot be used effectively as a sole fitness function (Fig. 3 ▶). Nonetheless, as seen in Fig. 4 ▶, the average skewness of the population seemed to improve in parallel with the average WRMS phase error during the two experiments. This discrepancy shows that the skewness is not a sufficiently specific target function by itself. This is consistent with previous studies showing that in low-resolution electron-density maps the electron-density histogram quality does not necessarily correlate with the phasing quality (Lunin *et al.*, 2012 ▶).

Our initial proof-of-concept study suggests multiple directions for further development and improvement. The first obvious area would be in extending the number of reflections (and consequently the resolution). Because our control experiment indicated that the GA had difficulty converging to good phase sets when the number of reflections was large, we anticipate that the most fruitful way of reaching higher resolution will be to begin at low resolution and then add reflections extracted from higher resolution bins. The ability of the GA approach to obtain phases by extension will be tested in future studies. In order to apply the procedure to real crystallographic problems, additional issues will have to be dealt with, including experimental measurement errors, the presence of a nonzero bulk-solvent density and dealing with unobserved low-resolution reflections.

Other improvements or variations could help to reduce the search space and the complexity of the task. For instance, as in Uervirojnangkoorn *et al.* (2013 ▶), instead of beginning with purely random phases we could choose initial phases for the strongest reflections according to probability distributions implied by preliminary experimental data (*e.g.* SIR). Another improvement could be made at the level of the Fourier transform implementation. For simplicity, electron-density maps are currently generated with a direct summation of the Fourier terms. Implementing an FFT (Ten Eyck, 1973 ▶) would substantially speed up the calculation of electron-density maps. For the current experiments, user selection rather than map calculation was limiting, but this could change at higher resolution. In the future, we would also like to assess the performance of GA variants within our framework. Those include the geographical niche theory (Li *et al.*, 2012 ▶), or adaptive approaches where genetic operators see their values vary along the process depending on the population variance (Ye *et al.*, 2010 ▶) or on probability matrices (Law & Szeto, 2007 ▶). As another variation on the human selection scheme, we envision that it might be useful for users to have the option to visualize enantiomeric or Babinet (*i.e.* negated) copies of individual maps while making their selections.

Finally, the user training could be improved. One way of improving the overall performance might be to define more player levels than the two initially implemented (0 and 1), so that greater control could be exerted over the use of player selections depending on their skill levels. These levels could also be used to control which players are able to advance to more complex puzzles or to restrict particular players to a limited number of tournament selections in each generation. In addition, in future work it will be important to train users on training problems that are different from the test problems (*i.e.* to use cross-validation) so that a truer sense can be gained about how the program can be expected to perform on truly unknown problems.

In summary, our work takes the first tentative steps towards ‘gamification’ of the phase problem, and lays the foundations for bringing crowdsourcing into the field of crystallography. Future developments should enable applications to more complex problems, including real blind cases where phases are not known. If those studies are successful, crowdsourcing could become a viable strategy for phasing in certain types of crystallographic problems.
